# Intrapartum Maternal Fever and Long-Term Infectious Morbidity of the Offspring

**DOI:** 10.3390/jcm12093329

**Published:** 2023-05-07

**Authors:** Omri Zamstein, Tamar Wainstock, Eyal Sheiner

**Affiliations:** 1 The Obstetrics and Gynecology Division, Soroka University Medical Center, Ben-Gurion University of the Negev, Beer-Sheva POB 151, Israel; 2 The Department of Public Health, Faculty of Health Sciences, Ben-Gurion University of the Negev, Beer-Sheva POB 653, Israel

**Keywords:** intrapartum fever, labor, childhood infections

## Abstract

Maternal intrapartum fever can lead to various maternal and neonatal complications and is attributed to various etiologies including infectious and non-infectious processes. In this study, we evaluated whether intrapartum fever affects the offspring’s tendency to long-term infectious morbidity. A population-based cohort analysis including deliveries between 1991 and 2021 was conducted. The incidence of hospitalizations of the offspring up to the age of 18 years, due to various infectious conditions, was compared between pregnancies complicated by intrapartum fever and those that were not. A Kaplan–Meier survival curve was used to assess cumulative hospitalization incidence. A Cox proportional hazards model was used to control for confounders. Overall, 538 of the 356,356 included pregnancies were complicated with fever. A higher rate of pediatric hospitalizations due to various infectious conditions was found among the exposed group, which was significant for viral, fungal and ENT infections (*p* < 0.05 for all). The total number of infectious-related hospitalizations was significantly higher (30.1% vs. 24.1%; OR = 1.36; *p* = 0.001), as was the cumulative incidence of hospitalizations. This association remained significant after controlling for confounders using a Cox proportional hazards model (adjusted HR = 1.21; 95% CI 1.04–1.41, *p* = 0.016). To conclude, fever diagnosed close to delivery may influence offspring susceptibility to pediatric infections.

## 1. Introduction

Intrapartum fever is defined as a maternal temperature of over 38.0 °C encountered during the active phase of labor, and delivery and can be attributed to various etiologies including infectious and non-infectious conditions, some of them readily resolvable. The reported prevalence of intrapartum fever ranges from 1 to 10% [1,2]. An important etiology of intrapartum fever is intraamniotic infection (IAI), which should be treated with broad-spectrum antibiotics, even without meeting the complete set of diagnostic criteria (including fetal tachycardia, maternal leukocytosis and purulent-appearing amniotic fluid) [3]. Prompt antimicrobial treatment, especially in the presence of other risk factors such as preterm labor or prolonged rupture of membranes, is advised by the American College of Obstetricians and Gynecologists and other international guidelines [4]. Another cause for intrapartum fever is neuraxial anesthesia [5,6], by a mechanism of inflammatory cytokine release [7]. Although neuraxial-related fever is considered benign, there is no practical way to distinguish between it and IAI, and these women are often treated with antibiotics empirically, to reduce risks for maternal and neonatal complications if IAI was be left untreated [4,8]. Other less common causes of temperature elevation include non-obstetric infectious processes (e.g., urinary tract infection) and fever associated with the administration of certain drugs.

Several studies have evaluated the consequences of intrapartum fever on the newborn [9,10,11]. Ongoing infection that causes maternal fever during labor can be vertically transmitted to the fetus and lead to neonatal sepsis, meningitis, or pneumonia shortly after birth [12]. Isolated fever of non-infectious etiology is also linked to adverse neonatal outcomes. A study including more than 3200 low-risk nulliparous women found that intrapartum maternal fever attributed to epidural analgesia was associated with a two-to-six-fold increased risk of adverse neonatal outcomes including hypotonia, assisted ventilation use, lower Apgar scores and neonatal seizures [13]. The suggested mechanism is the release of maternal cytokines [14], such as IL-6 [15], that translocate to the fetal circulation and act as mediators of injury [16]. The implications of liberal antibiotic use during labor, the infectious process itself or the pro-inflammatory environment associated with elevated body temperature, may extend beyond the neonatal period, although the long-term significance remains largely unknown. In this study, we sought to evaluate whether intrapartum fever poses a risk for long-term infectious morbidity of the offspring throughout childhood.

## 2. Materials and Methods

This population-based cohort analysis included singleton pregnancies of women who delivered during the period 1991–2021 at the Soroka University Medical Center (SUMC), a tertiary hospital in Israel serving a population of over 1 million residents. The institutional review board at Soroka University Medical Center approved this study in accordance with the Helsinki Declaration’s ethical standards. The primary exposure was defined as a diagnosis of intra-partum fever (i.e., two temperature readings equal to or greater than 38.0 °C during the active phase of labor); outcomes included hospitalization of the corresponding offspring due to preselected infectious conditions, pre-defined according to the International Classification of Diseases 9 until 18 years of age. Multiple gestations and cases of fetuses with major structural anomalies were excluded from the analysis, to avoid potential confounding posed by these unique pregnancies [17]. Data comprised two sets of databases; the first is the Obstetrics and Gynecology Department database, which includes perinatal information documented shortly after each delivery by the attending obstetrician and is then thoroughly reviewed by designated secretaries. The second is the SUMC ICD-9 database, comprising demographic characteristics and diagnoses given during medical encounters within the hospital. These two databases are merged at the individual level.

Data were analyzed using SPSS version 26.0 for Windows. Categorical variables were described using frequencies and numerical distributions, and continuous variables that are normally distributed were described using mean and standard deviation. The differences between the groups were assessed using two-sample *t*-test, Mann–Whitney test, or chi-squared test in accordance with variable type and its distribution. Kaplan–Meier survival curves were built to determine the cumulative incidence of infectious-related hospitalizations, and the Cox–Mantel log-rank was used to compare the groups. Cox proportional hazards model was applied to control for clinically relevant and significant confounders. All analyses were two-sided, and *p*-values of less than 0.05 were considered significant.

## 3. Results

During the study period, 356,356 pregnancies met the inclusion criteria; 538 were complicated with intrapartum fever. Parturients with fever were more likely to be nulliparous and in preterm labor. Fever was also more common with the use of epidural labor analgesia. Almost half of the cases of intrapartum fever were associated with labor induction. Intrapartum fever was linked to abnormal fetal heart rate patterns, cesarean delivery (CD), and lower Apgar scores at 5 min. More cases of perinatal mortality were observed when fever complicated delivery (*p* < 0.001 for all; Table 1).

**Figure 1:**
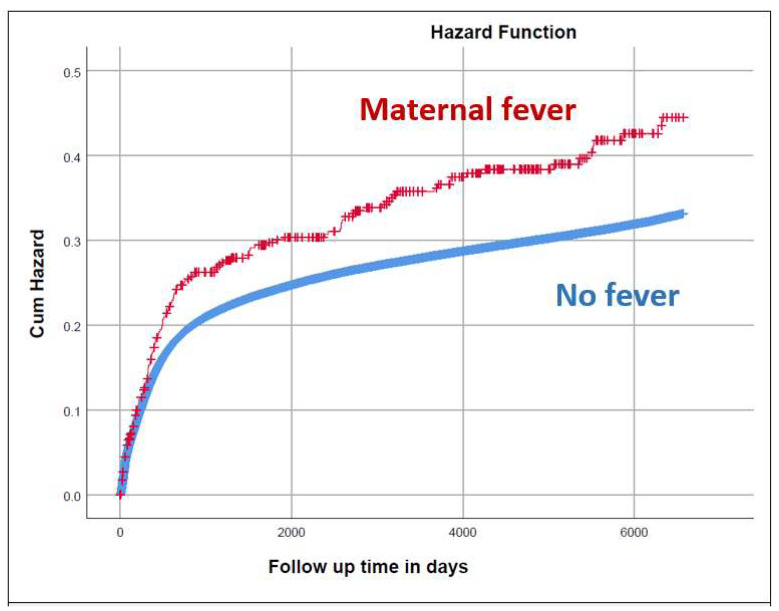
Cumulative incidence of hospitalization with infectious morbidity in offspring according to maternal peripartum fever. Log rank *p*-value = 0.001.

As for the long-term outcomes of the offspring, a higher rate of pediatric hospitalizations due to various infectious conditions was found, which was significant for viral, fungal and ENT infections (*p* < 0.05; Table 2). The total number of infectious-related hospitalizations was significantly higher among the exposed group (30.1% vs. 24.1%; OR = 1.36; 95% CI 1.13–1.64, *p* = 0.001), as was the cumulative incidence of hospitalizations presented in the Kaplan–Meier survival curve (log-rank *p*-value = 0.001; Figure 1). The association between fever during delivery and infectious-related hospitalization of the offspring remained significant after controlling for selected confounders (e.g., gestational age, maternal GBS colonization, cesarean delivery, non-reassuring fetal heart rate patterns and diabetes mellitus) using a Cox proportional hazards model (adjusted HR = 1.21; 95% CI 1.04–1.41, *p* = 0.016; Table 3).

## 4. Discussion and Conclusions

The primary study objective was to assess the impact of maternal fever during labor on the long-term infectious morbidity of the offspring. Our findings suggest that intrapartum temperature elevation from various etiologies is linked to unfavorable obstetric outcomes including non-reassuring fetal heart rate patterns and emergent CD. It is also independently associated with long-term hospitalization of the offspring due to various infectious conditions, including viral, fungal and ENT infections.

The relatively low prevalence of intrapartum fever in our cohort can be related to a prudent definition of fever in our practice, which requires two separate temperature readings. Since there are no formal guidelines on when and how (e.g., oral, rectal) to measure maternal temperature during labor, additional fever cases were possibly missed. As expected, nulliparity and labor induction due to various medical indications (e.g., oligohydramnios [18], pregnancy following fertility treatments [19]) are associated with the slow progress of labor [20,21,22], which increases the risk for pathogenic vaginal flora migration into the amniotic cavity and development of intra-partum fever [23,24,25]. The association between fever during labor and category II fetal heart rate tracing has also been described previously [26]. The fetal heart rate abnormality primarily associated with infection is fetal tachycardia, due to increased metabolic rate and oxygen consumption. Progressive stages of cardiogenic- and septic-related circulatory depression of the fetus due to infection can eventually lead to reduced variability and periodic decelerations of fetal heart rate [27]. Even though intra-amniotic infection alone is not an indication for CD, clinicians’ efforts to hasten delivery in the presence of fetal distress [28] may have accounted for the higher CD rates observed in our study population [29]. Both preterm and term infants infrequently succumb to the infectious process [30].

While maternal fever is known as an important predictor of neonatal infection-related morbidity and mortality [1], evidence for a link between intrapartum fever and future infectious morbidity is limited. Many pediatric disorders during the first few years of life, for example, malnutrition, hematologic malignancy and chronic disease [31,32], can lead to a transient or continuous state of acquired immunodeficiency and thus predispose offspring to recurrent infections.

We postulate that the tendency towards higher infection rates among children and infants in our study is attributed to several factors. First is the sequela of the initial infection, for example, chorioamnionitis complicated by neonatal pneumonia [33], which in turn impairs lung function [34] and results in increased vulnerability to repeat infections [35]. In addition, prematurity (seen more commonly among the intrapartum fever group) is itself strongly linked to recurrent infections [36,37,38]; previous publications from our institution found an inverse relationship between the birth week of the premature infant and the risk for infectious morbidity [38], highlighting the process of immune system maturation that takes place throughout gestation [39]. Fetal inflammatory response syndrome (FIRS) triggered by elevated maternal temperature can also play a contributory role, even in the absence of proven infection [40,41]. Fetuses with FIRS have been shown to suffer from derangements in various body systems, including myocardial dysfunction [42], chronic lung injury [43], altered hematologic profile with higher neutrophil count [44] and thymus size reduction [45], all of which can adversely impact their transition to extra-uterine life. Furthermore, although broad-spectrum antibiotics is the cornerstone of treatment for suspected intraamniotic infection, in utero and postnatal exposure [46] to antimicrobial agents may also alter host natural defense mechanisms and contribute to later pediatric morbidity [47]. We have revealed statistically significant differences for some types of infections (e.g., viral, ENT) that are generally more common in the pediatric population [48,49,50]. The lack of statistical significance of the rest may be explained by the small differences in the incidence between the study groups and the insufficient power to detect the differences.

Acetaminophen is commonly used in our institution as part of the bundle of treatment for clinical chorioamnionitis. Although it is reported to correct fetal heart rate patterns and acid–base status, there is no clear evidence of it improving neonatal outcomes [28]. Limited access to medical care and lack of breastfeeding, are linked to both acute and chronic illnesses among children [51]. Lower breastfeeding rates among patients undergoing CD [52] or who experience fever during labor may have influenced the outcomes.

The limitations of our study are intrinsic to the methodology, including the inability to infer causality between fever exposure during delivery and long-term infectious morbidity, limited validation process of clinical records and different hospitalization standards throughout the years. Moreover, the cause of maternal fever for which we could not account can impact neonates’ health differently. It is also impractical to differentiate the direct effect of fever from that of the underlying cause [53]. Due to database considerations, we had no information regarding other factors that may influence susceptibility to infectious morbidity, such as prenatal steroid treatment, duration of rupture of membranes and antibiotic treatment initiation. However, we do comply with international guidelines and tend to treat upon suspicion of IAI. Finally, hospitalization serves only as a surrogate of childhood morbidity, as many childhood infections are treated in ambulatory settings.

The strengths of our study include the large sample sizes, high rates of follow-up and good generalizability owing to a well-balanced representation of this area’s population [54]. Taken together, our study provides support for the influence of exposure to fever during labor and long-term offspring’s health. Any short- and long-term complications may be due to the fever or the cause of the fever. The extent to which early diagnosis and prompt treatment of intrapartum fever may influence neonatal and childhood outcomes should serve as the basis of future studies.

## Figures and Tables

**Table 1:** Demographical, obstetrical and perinatal characteristics of pregnancies with and without peripartum fever (PPF).

Characteristic	Peripartum Fever (*n* = 538)	No Peripartum Fever (*n* = 355,818)	*p*-Value ^a^
Maternal age at index birth (years ± SD)	27.2 ± 5.5	28.3 ± 5.8	<0.001
Primiparity (%)	53.3	24.1	<0.001
Maternal diabetes mellitus (%)	5.9	4.8	0.201
Hypertensive disorders of pregnancy (%) ^b^	6.3	4.7	0.071
Pregnancy following fertility treatments (%) ^c^	3.2	1.7	0.007
Mean gestational age (weeks ± SD)	38.4 ± 3.1	39.0 ± 2.0	<0.001
Epidural labor analgesia	43.1	19.6	<0.001
Preterm delivery (<37 weeks, %)	13.4	6.9	<0.001
IUGR (%) ^d^	1.3	1.7	0.43
Oligohydramnios (%)	3.5	2.2	0.036
Prelabor rupture of membranes (%)	3.3	0.5	<0.001
Induction of labor (%)	45.7	21.2	<0.001
Maternal GBS colonization (%)	0.1	0.8	1.0
Non-reassuring fetal heart rate patterns (%)	25.8	5.3	<0.001
Meconium-stained amniotic fluid (%)	24.7	12.0	<0.001
Placental abruption (%)	1.1	0.5	0.065
Prolonged second stage of labor (%)	4.5	1.2	<0.001
Cesarean delivery (%)	36.4	14.0	<0.001
Apgar score < 7 at 5 min (%)	2.5	0.6	<0.001
Birthweight (grams ± SD)	3153.0 ± 649	3200.4 ± 519	0.09
Cord blood pH < 7.0 (%)	6.7%	0.4%	0.059
Low birth weight ≤ 2500 g (%)	10.0	6.9	0.004
Very low birth weight ≤ 1500 g (%)	3.3	0.8	<0.001
SGA (%) ^e^	2.0	4.6	0.05
Perinatal mortality (%)	2.8	0.8	<0.001

^a^ Calculated using X2 test for trends. ^b^ Including chronic hypertension, gestational hypertension and preeclampsia. ^c^ Ovulation induction or in vitro fertilization. ^d^ Intrauterine growth restriction defined as estimated fetal weight less than the 10th percentile for gestational age. ^e^ Small for gestational age defined as less than the 5th percentile for gestational age.

**Table 2:** Infectious hospitalization of the offspring by peripartum fever status.

Infectious Condition	Peripartum Fever (*n* = 538)	No Peripartum Fever (*n* = 355,818)	*p*-Value
Bacterial infections (%)	2.0	1.5	0.26
Viral infections (%)	5.2	3.2	0.009
Fungal infections (%)	0.4	0.1	0.045
CNS infections ^a^ (%)	0.7	0.5	0.37
ENT infections ^b^ (%)	8.6	6.4	0.044
Respiratory infections (%)	16.5	13.8	0.067
Gastrointestinal infections (%)	4.3	3.0	0.07
Total hospitalizations (%)	30.1	24.1	0.001

^a^ Central nervous system infections; ^b^ Ear, nose and throat infections.

**Table 3:** Cox hazards regression model for the factors associated with infectious hospitalization of the offspring.

Characteristic	HR	95% CI	*p*-Value
Maternal fever	1.21	1.04–1.41	0.016
Gestational age (weeks)	0.95	0.94–0.95	<0.001
Maternal age at birth	0.99	0.98–1.0	<0.001
Maternal diabetes mellitus ^a^	1.07	1.04–1.11	<0.001
Hypertensive disorders of pregnancy ^b^	1.04	1.01–1.07	0.02
Maternal GBS ^d^ colonization	1.21	1.12–1.32	<0.001
Non-reassuring fetal heart rate patterns	1.04	1.01–1.07	0.008
Cesarean delivery	1.11	1.09–1.13	<0.001
Very low birth weight ≤ 1500 g	0.59	0.54–0.65	<0.001

^a^ Including gestational and pregestational diabetes mellitus; ^b^ Including chronic hypertension, gestational hypertension and preeclampsia; ^d^ group b streptococcus.

## Data Availability

Data sharing is not applicable to this article.
